# Imaging features of pancreatic extragastrointestinal stromal tumors: a case report and literature review

**DOI:** 10.3389/fonc.2025.1638850

**Published:** 2025-07-29

**Authors:** Jian-Xia Xu, Meng-Yin Gu, Yi-Hua Wang, Xiao-Shan Huang, Jia-Qi Duan, Xiao-Zhong Zheng, Xi-Xi Chen, Ri-Sheng Yu, Jun-Yi Xiang

**Affiliations:** ^1^ Department of Radiology, The Second Affiliated Hospital of Zhejiang Chinese Medical University, Hangzhou, Zhejiang, China; ^2^ Department of Radiology, The First People’s Hospital of Wenling, Wenling, Zhejiang, China; ^3^ Department of Radiology, Second Affiliated Hospital, School of Medicine, Zhejiang University, Hangzhou, Zhejiang, China

**Keywords:** pancreas, extragastrointestinal stromal tumors, literature review, case report, imaging features

## Abstract

Pancreatic extra-gastrointestinal stromal tumors (pEGISTs) are exceedingly rare, accounting for less than 5% of all extra-gastrointestinal stromal tumors (EGISTs). We report a case involving a 62-year-old woman who presented with a hypervascular pancreatic mass. Postoperative histopathological examination confirmed the diagnosis of a gastrointestinal stromal tumor (GIST). The neoplastic cells exhibited a spindle-shaped morphology without evident necrosis on histologic sections. Immunohistochemical analysis demonstrated strong, diffuse positivity for CD117, DOG-1, and CD34. Notably, the imaging characteristics of this pEGISTs displayed several distinctive features, including intralesional “island-like” enhancement, peripheral nodular enhancement, and prominent vascular “staghorn” patterns. Although histopathology remains the gold standard for definitive diagnosis of pEGISTs, correlating clinical presentation with these unique radiologic features can substantially improve diagnostic accuracy for radiologists. Additionally, we provide a comprehensive review and discussion of the current literature on the clinical and imaging hallmarks of pEGISTs.

## Introduction

Gastrointestinal stromal tumors (GISTs) are mesenchymal neoplasms arising from the interstitial cells of Cajal within the gastrointestinal (GI) tract and are recognized for their potential malignant behavior (1). These tumors can develop anywhere along the GI tract, with the stomach and small intestine being the most frequently involved sites. Extra‐gastrointestinal stromal tumors (EGISTs) are defined as tumors that share identical histopathological and immunohistochemical profiles with GISTs but originate outside the GI tract, including locations such as the retroperitoneum, momentum, mesentery, or pancreas (2). EGISTs are uncommon, accounting for only 5%–10% of all GIST cases (1, 3–5). (pEGISTs are exceedingly rare; to date, only sporadic case reports have been described in both Chinese and English literature, and no systematic investigations have specifically characterized their imaging features (6). Given the lack of distinctive clinical manifestations, preoperative diagnosis is particularly challenging, and pEGISTs are often misdiagnosed as pancreatic neuroendocrine neoplasms (pNENs), solid pseudopapillary neoplasms (SPNs), mucinous cystic neoplasms (MCNs), or pancreatic pseudocysts.

In this study, we present a rare case of pEGISTs, providing a comprehensive description of its clinical presentation and imaging characteristics. We also systematically review recent Chinese and English literature on pEGISTs, with particular emphasis on studies that include computed tomography (CT) imaging. By summarizing the clinical and radiologic features of these tumors, we aim to enhance diagnostic accuracy among radiologists and inform clinical decision‐making, thereby improving therapeutic outcomes and patient prognosis.

## Results

### Case presentation

A 62-year-old woman underwent a routine health examination that incidentally revealed a pancreatic mass. She denied any abdominal pain or other discomfort but reported an unintentional weight loss of approximately 2.5 kg over the preceding six months. The outpatient department provisionally diagnosed her with a “pancreatic tumor,” and she was admitted for further evaluation. On physical examination, mild tenderness was elicited in the upper abdomen without rebound tenderness or guarding, and a palpable mass was noted in the epigastric region.

On admission, laboratory investigations—including complete blood count, liver and renal function tests, electrolytes, and tumor markers (CA19-9, CEA)—were all within normal limits. Her past surgical history included a cesarean delivery 28 years ago and a subtotal hysterectomy 23 years ago. Her medical history was notable for hypertension of over ten years’ duration (well controlled on oral antihypertensive medication), type 2 diabetes mellitus for approximately one year (well controlled on oral hypoglycemics), and asthma for more than ten years (currently not on any medications).

**Figure 1:**
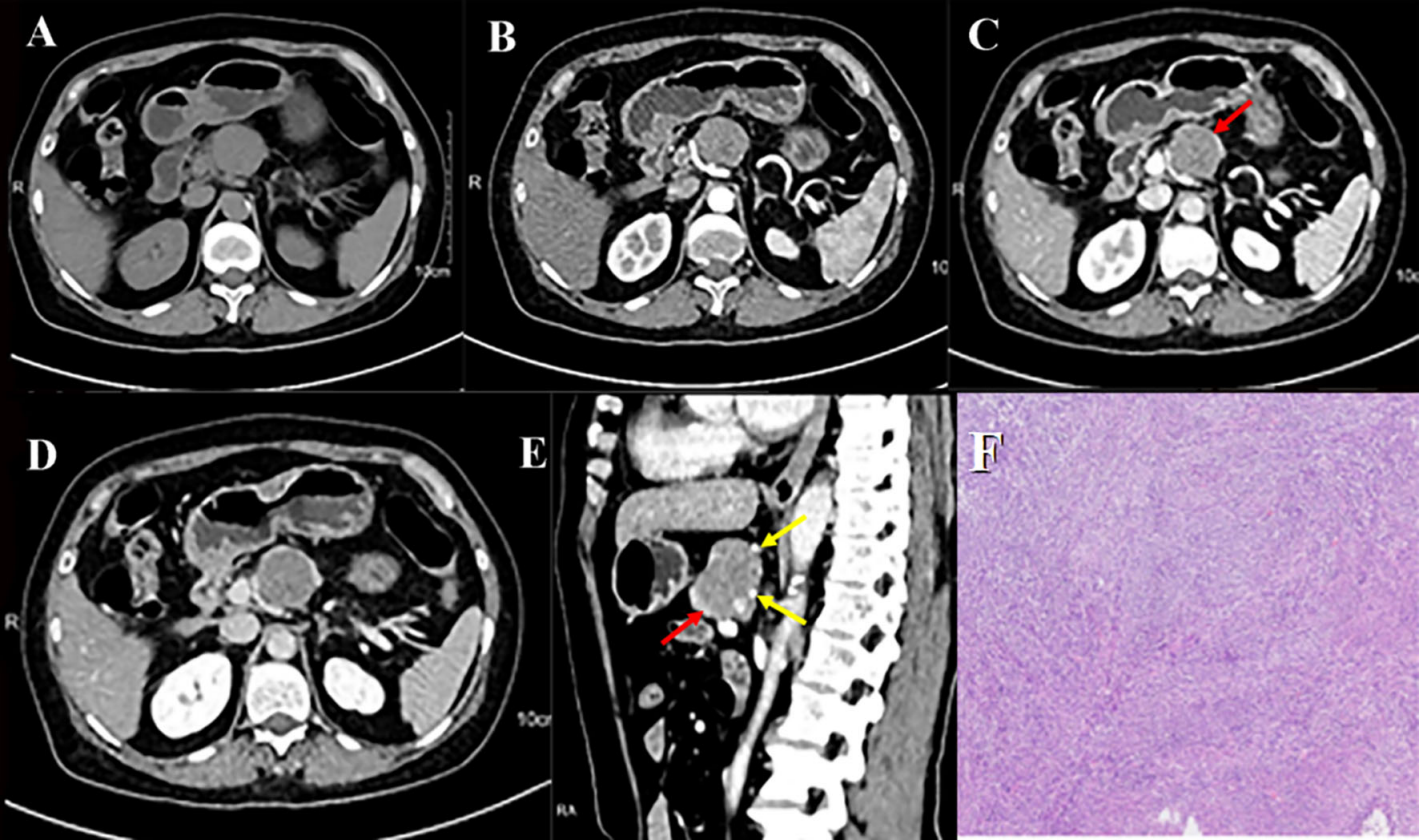
pEGISTs, pancreatic CT scan shows multiple-phase enhancement with marginal enhancement and Perilesional vascular sign. Axial unenhanced **(A)** reveals an isodense mass in the body of the pancreas; arterial **(B)**, portal venous **(C)**, and equilibrium **(D)** phases show moderate enhancement with marginal enhancement signs (red arrow); sagittal enhanced **(E)** shows both marginal enhancement (red arrow) and Perilesional vascular sign (yellow arrow). **(F)** Histological examination of pathological biopsy showed spindle cell tumor in the pancreatic body. The tumor was mainly composed of spindle cells. Original magnification: ×100.

**Figure 2:**
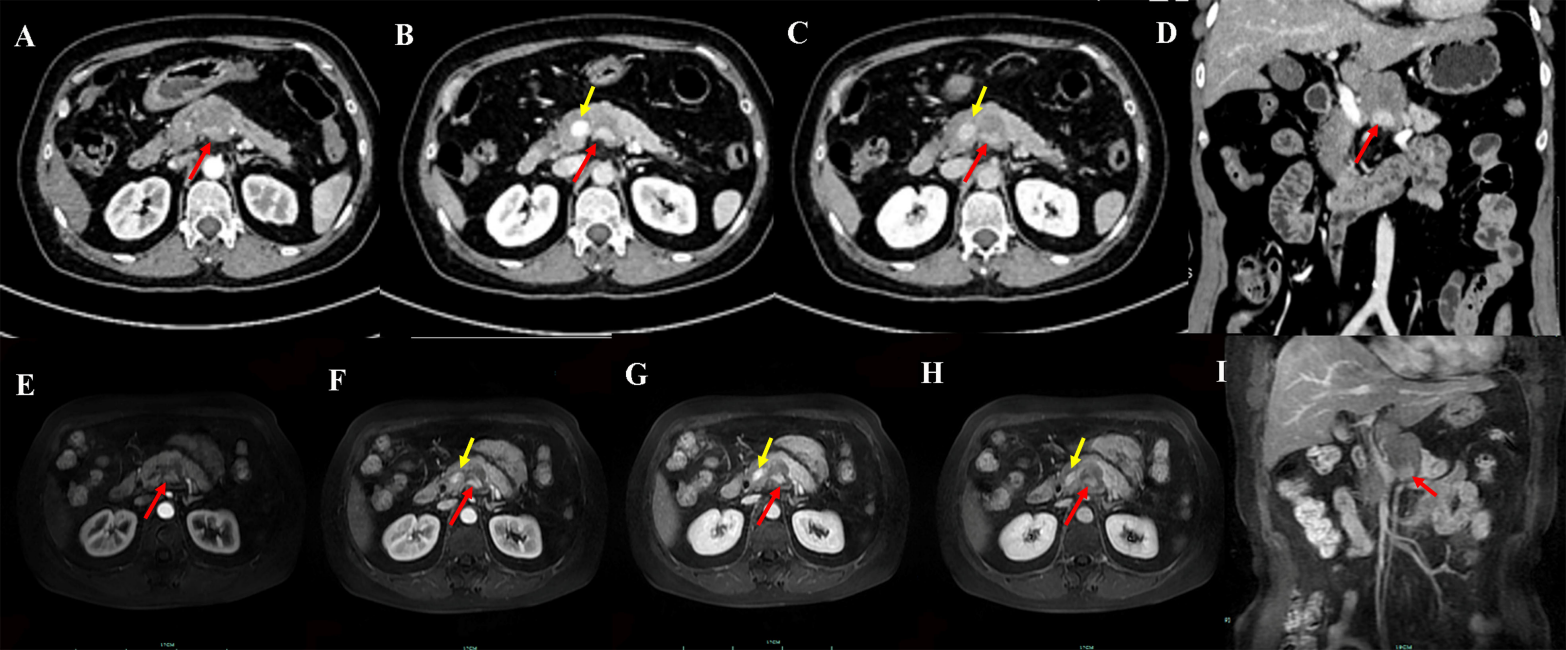
Pancreatic CT multi-phase enhancement in axial **(A-C)** and coronal **(D)** views shows a nodular area with more prominent enhancement, referred to as ‘island-like enhancement’ (red arrow), and enhanced portal vein (yellow arrow). Pancreatic MR multi-phase enhancement in axial **(E-H)** and coronal **(I)** views shows ‘island-like enhancement’ (red arrow) and enhanced portal vein (yellow arrow).

Contrast-enhanced computed tomography (CT) of the pancreas revealed a well-circumscribed, round, isoattenuating mass in the pancreatic body, measuring approximately 3.2 cm × 3.6 cm × 3.7 cm. On non-contrast imaging, the lesion’s attenuation measured 41.8 HU. Following contrast administration, the mass exhibited heterogeneous, moderate enhancement, with attenuation values of 55.9 HU in the arterial phase, 68.7 HU in the portal venous phase, and 56.9 HU in the delayed phase (**Figures 1A–D**; **Supplementary Figure S1**). Of particular note, the periphery of the lesion displayed nodular, “rim-like” enhancement (hereafter referred to as the “marginal enhancement sign”), and engorged vessels were seen at the lesion margin (the “Perilesional vascular sign,” **Figures 1D, E**). **Figure 1** demonstrates a well-circumscribed hypervascular pancreatic mass(A-D) with marginal enhancement (D-E, red arrows) and perilesional vascular signs (E, yellow arrows), typical imaging features of pEGISTs. A nodular area of intense enhancement was observed within the lesion, referred to as the “island-like enhancement sign,” with attenuation values of 106 HU, 140 HU, and 115 HU in the arterial, portal venous, and delayed phases, respectively (**Figures 2A–D**). **Figure 2** highlights this feature on both CT and MRI, where nodular intratumoral areas demonstrate stronger enhancement than surrounding tissue (red arrows), suggesting viable tumor components within a heterogeneous matrix. Yellow arrows indicate adjacent portal vein enhancement. Upstream, mild atrophy of the pancreatic parenchyma and slight dilation of the main pancreatic duct were observed. The splenic artery was displaced posteriorly by compression from the mass, while the surrounding fat planes remained clear, with no evidence of adjacent vascular invasion. No significantly enlarged lymph nodes were identified in the retroperitoneum. Based on these findings, a neoplastic process was favored, with the leading preoperative differential diagnoses being pancreatic neuroendocrine neoplasm (pNEN) or solid pseudopapillary neoplasm (SPN).

**Figure 3:**
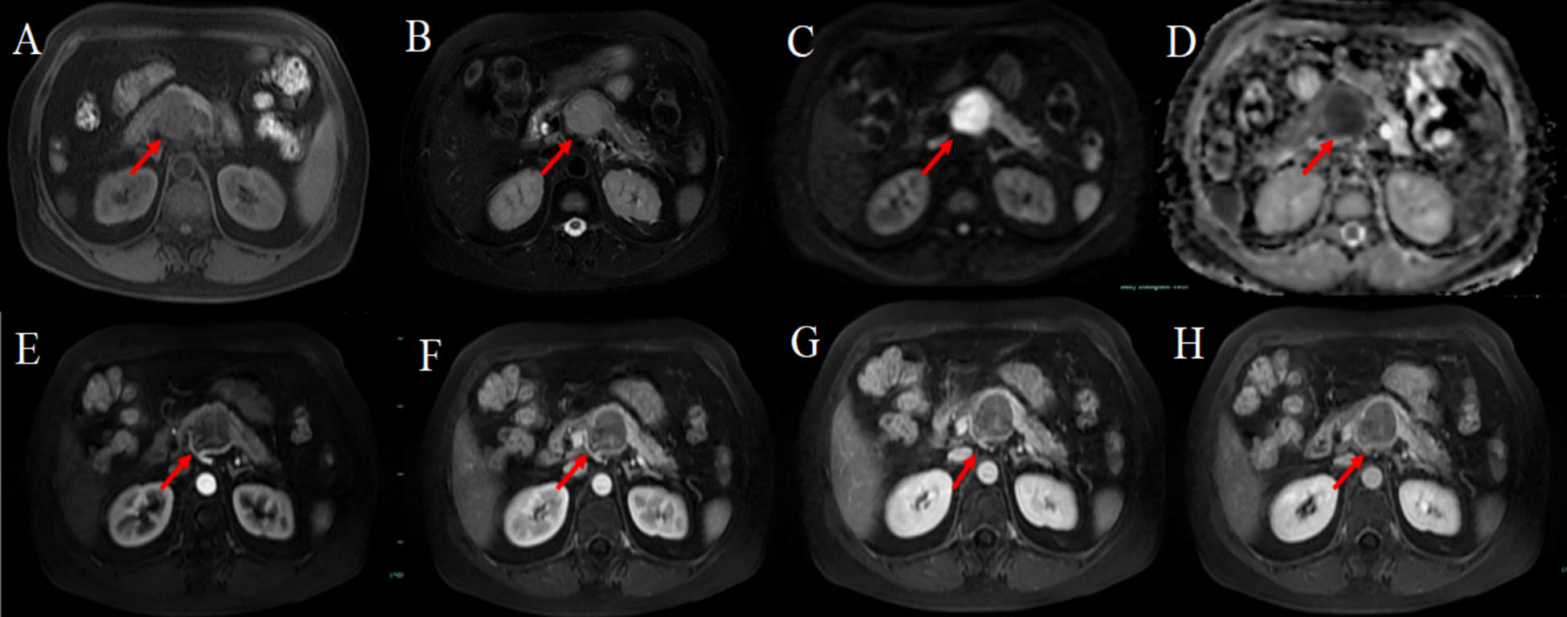
MRI axial unenhanced **(A)** fat-suppressed T1WI shows the lesion (red arrow) as low signal, and fat-suppressed T2WI shows high signal; **(C)** DWI shows high signal, **(D)** ADC mapping shows low signal; axial multi-phase enhancement **(E-H)** shows the lesion with mild enhancement, marginal enhancement signs, and faint island-like enhancement signs.

Magnetic resonance imaging (MRI) of the pancreas further characterized the lesion. On T1-weighted imaging (T1WI), the mass was hypointense, while on T2-weighted imaging (T2WI) it appeared uniformly mildly hyperintense, with well-defined margins. Diffusion-weighted imaging (DWI) demonstrated marked hyperintensity, and the corresponding apparent diffusion coefficient (ADC) map showed substantially reduced signal, indicating restricted diffusion (**Figures 3A–D**). Following multiphase contrast enhancement, the lesion exhibited heterogeneous moderate enhancement (**Figures 3E–H**), with nodular foci of more intense enhancement observed within the mass (**Figures 2E–I**). **Figure 3** illustrates the MRI features of the pancreatic lesion.

Under general anesthesia, the patient underwent a laparoscopic distal pancreatectomy with splenectomy and peripancreatic neurolysis. Gross examination of the resected specimen and adjoining splenic tissue was followed by histopathologic analysis, which revealed a spindle-cell neoplasm composed of densely packed cells exhibiting mild atypia(**Figure 1F**).Immunohistochemistry showed the following profile: CK-PAN (–), EMA (–), CD34 (+), S100 (focally +), Desmin (–), Ki-67 (3%+), STAT6 (–), β-catenin (–), α-SMA (focally +), MDM2 (scant +), SS18-SSX (–), NKX2.2 (–), CD99 (focally +), pan-TRK (weak +), DOG1 (+), CD117 (+), SOX10 (–), H3 K27me3 (focally +), GLI-1 (–), WT1 (scant +), BCOR (–), SDHB (positive expression), Synaptophysin (–), Chromogranin A (–), INSM1 (–), Calretinin (–), GATA3 (occasional +), CDK4 (focally +), CD21 (–), CD23 (–), and CD35 (–). Molecular genetic testing for KIT gene exons 9, 11, 12, 13, 14, 17, 18, and PDGFRA gene exons 12, 14, and 18 showed no detectable mutations. Taken together, the histologic, immunophenotypic, and molecular findings supported a diagnosis of a borderline or low‐malignant‐potential spindle‐cell neoplasm, consistent with a wild‐type gastrointestinal stromal tumor (wild-type GIST).

### Review of literature

A literature search identified 76 published articles on pancreatic EGISTs (pEGISTs). Of these, 48 studies (encompassing 50 patients) included CT imaging data and were thus selected for detailed analysis. We extracted and aggregated clinical and imaging characteristics for all 50 patients (**Supplementary Table S1**; **Table 1**). The median age at diagnosis was 55 years (range: 30–74 years), with a nearly equal sex distribution (24 females [52%] and 26 males [48%]). The most common tumor location was the pancreatic head (22 cases, 44%), followed by the pancreatic body–tail region (11 cases, 22%). Tumor size ranged from 2.0 cm to 35.0 cm in maximum diameter, with a mean size of 10.2 cm.

**Table 1:** Analysis of clinical and imaging features of pEGISTs.

Features	N	Percentage
Age		
≤55	26	52%
>55	24	48%
Sex		
Male	26	52%
Female	24	48%
Tumor location		
Body	4	8.0%
Body+Tail	11	22%
Head	22	44%
Head+Uncinate	2	4%
Tail	8	16%
Uncinate	3	6%
Tumor size(cm)		
≤5	16	32%
>5	34	68%
Component		
Solid	22	44%
Solid-cystic	13	26%
cystic -solid	14	28%
cystic	1	2%
Margin		
well-defined	25	50%
Partially ill-defined	25	50%
Shape		
Round	22	44%
Oval	15	30%
Irregular	13	26%
calcification		
No	20	40%
Yes	1	2%
NA	29	58%
Necrosis		
No	11	22%
Yes	39	78%
Enhancement types		
heterogeneous	46	92%
homogeneous	4	8%
Enhancement degree		
mild	20	40%
moderate	2	4%
marked	28	56%
Perilesional vascular sign		
No	14	28%
Yes	36	72%
Island-like enhancement sign		
No	36	72%
Yes	14	28%
Marginal enhancement sign		
No	24	48%
Yes	26	52%
Imaging misdiagnosed lesions		
NA	27	54%
Malignant lesion	6	12%
Pseudocyst	2	4%
Benign lesion	1	2%
pNENs	6	12%
SPN	4	8%
PDAC	1	2%
MCN	2	4%
Duplication cyst	1	2%
NIH risk		
High	29	58%
Intermediate	6	12%
Low	7	14%
NA	8	16%

Solid-cystic, Predominantly solid with cystic component.

cystic -solid, Predominantly cystic with peripheral solid component.

pNENs, Neuroendocrine tumors.

SPN, Solid pseudopapillary neoplasm.

MCN, Mucinous cystic neoplasms.

PDAC, Pancreatic ductal adenocarcinoma.

NIH, National institutes of health.

NA, Unable to assess.

On CT imaging, 27 lesions (54%) appeared predominantly cystic‐solid (14 cystic‐predominant; 13 solid‐predominant), 22 lesions (44%) were purely solid, and only one lesion (2%) was purely cystic. Among the 16 tumors measuring ≤ 5 cm in diameter, 12 (75%) manifested as solid masses, three (19%) exhibited solid‐predominant cystic‐solid change, and one (6%) was predominantly cystic. In contrast, of the 34 tumors > 5 cm, cystic or cystic‐solid morphology predominated (24 cases, 70.6%). Tumor margins were clear in most cases; however, lesions with borderline or overtly malignant potential occasionally demonstrated ill‐defined borders. Morphologically, tumors were round in 22 cases (44%), oval in 15 cases (30%), and irregular in 13 cases (26%). Necrosis was a frequent finding (39 cases, 78%), whereas calcification was rare (1 case, 2%). On contrast-enhanced CT, 46 lesions (92%) exhibited heterogeneous enhancement. Enhancement intensity was most commonly described as marked or mild, with moderate enhancement being less frequent. Notably, some lesions demonstrated characteristic imaging signs, including “island-like enhancement,” “marginal enhancement,” and a “Perilesional vascular sign.”

Among all reported cases in our review, none were correctly diagnosed preoperatively. Of 23 cases with documented preoperative misdiagnoses, six (26%) were incorrectly classified as malignant lesions of undetermined origin, six (26%) as pNENs, four (18%) as SPNs, two (9%) as MCNs, two (9%) as pseudocysts, one (4%) as pancreatic adenocarcinoma, one (4%) as a duplication cyst, and one (4%) as a benign cystic lesion.

## Discussion

GISTs represent the most common mesenchymal neoplasms of the gastrointestinal tract, arising from interstitial cells of Cajal or their precursors. Since GISTs were first delineated as a distinct pathological entity in 1983, they have become a focal point of research in gastrointestinal oncology. Their characteristic histological morphology and immunohistochemical profile—most notably strong expression of CD117 (KIT) and DOG1—provide a clear diagnostic framework (1). However, tumors with morphologic and immunophenotypic features indistinguishable from gastrointestinal GISTs but occurring outside the gastrointestinal tract, without anatomical continuity to the bowel wall or serosa, are classified as EGISTs. EGISTs account for only 5–10% of all GISTs, reflecting their relative rarity (2). The majority of EGISTs originate in mesenteric, omental, retroperitoneal, abdominal wall, hepatic, or pancreatic locations, with pEGISTs comprising less than 5% of cases (1–5). Because the pancreas is an uncommon site for EGISTs, pEGISTs are exceedingly rare; most published data derive from isolated case reports or small retrospective series, and a unified understanding of their clinical and radiologic features remains lacking.

The cellular origin of pEGISTs are not yet fully elucidated, although the prevailing hypothesis posits that they derive from ectopic interstitial cells of Cajal or undifferentiated mesenchymal precursors located in the peri-pancreatic or retroperitoneal region, which subsequently undergo stromal differentiation. Similar to gastrointestinal-type GISTs, pEGISTs lack specific clinical manifestations: early lesions are often asymptomatic, and many tumors are discovered incidentally during routine health examinations or imaging studies. Symptomatic patients may present with abdominal pain, discomfort, weight loss, distension, or an abdominal mass (6–54). Our present findings corroborate these clinical features (**Supplementary Table S1**). In our cohort of 50 patients, the median age was 55 years; 24 (48%) were female and 26 (52%) were male. The head of the pancreas was the most frequently involved site (22/50, 44%), followed by the body and tail (11/50, 22%). This distribution is consistent with earlier reports, which predominantly describe pEGISTs in middle-aged and elderly patients, with predilection for the pancreatic head and tail (55), and the gender distribution aligns with findings by Gupta and colleagues (55). Tumor sizes ranged from 2.0 to 35.0 cm, with a mean maximum diameter of 10.2 cm—again in agreement with prior literature, which attributes large tumor volumes at diagnosis to the insidious growth of pEGISTs and the absence of early, specific symptoms. Consequently, most patients have sizeable lesions at presentation that often compress or infiltrate adjacent structures (15). Consistent with our series and the reviewed literature, serum tumor markers such as CA19–9 and CEA were uniformly negative, indicating minimal utility of traditional serum markers in diagnosing pEGISTs (7–25).

Radiologic imaging is central to preoperative assessment of pEGISTs and can yield important diagnostic clues. Modalities include abdominal ultrasound, contrast-enhanced computed tomography (CT), magnetic resonance imaging (MRI), endoscopic ultrasound–guided fine-needle aspiration (EUS-FNA), and positron emission tomography–CT (PET-CT), with CT and MRI being paramount. However, previous publications have predominantly described CT features, with fewer reports of MRI characteristics; thus, a comprehensive summary of imaging findings is still needed. In our study, the MRI characteristics of the index case paralleled CT findings: on unenhanced sequences, the lesion exhibited low signal intensity on T1-weighted imaging (T1WI), high signal intensity on T2-weighted imaging (T2WI), and diffusion restriction on diffusion-weighted imaging. We systematically reviewed and summarized CT imaging features from 50 pEGISTs lesions. Although pEGISTs most frequently occur in the pancreatic head and body/tail regions, our illustrative case arose in the pancreatic body, a less common location. We stratified lesions by maximum diameter into ≤5 cm and >5 cm subgroups and analyzed their solid versus cystic composition. Among patients with lesions ≤5 cm (n = 16), 75% (12/16) were predominantly solid, 19% (3/16) were predominantly solid with patchy cystic change, and only 6% (1/16) were predominantly cystic. In contrast, patients with lesions >5 cm (n = 34) exhibited a higher proportion of cystic or mixed cystic-solid masses—70.6% (24/34). pEGISTs are hypervascular neoplasms, and their enhancement patterns on contrast-enhanced CT have the following salient features: (1) heterogeneous enhancement: 92% (46/50) in our series demonstrated heterogeneous enhancement, whereas 8% (4/50)—including one entirely cystic lesion—exhibited homogeneous enhancement. Marked heterogeneity is especially diagnostically suggestive. (2) Marginal enhancement sign and Perilesional vascular sign: 52% (26/50) showed Marginal enhancement sign. The “Perilesional vascular sign,” previously described in the literature (7–13), was observed in 72% of cases in our review, and we herein formally designate it as the Perilesional vascular sign. Combined Marginal enhancement sign and Perilesional vascular sign occurred in 40% of cases. Marginal enhancement sign on contrast-enhanced CT or MRI is characterized by pronounced enhancement of the tumor periphery with lack of central enhancement. This is typically due to central necrosis or cystic degeneration in GISTs resulting from rapid growth, leading to insufficient blood supply and minimal or absent enhancement centrally; in contrast, the proliferative, active tumor regions are often located at the periphery, where vascularity is rich and contrast permeability is high, producing marked rim enhancement. In addition, some GISTs develop a pseudocapsule with an abundant vascular network, which also manifests as a ring-like enhancement. The Perilesional vascular sign on contrast-enhanced CT or MRI appears as enhancing vessels at the tumor margin (whether dilated or not). This arises because pancreatic GISTs often receive blood supply from the peripancreatic arterial network or mesenteric collateral vessels; tumor growth can stimulate angiogenesis in adjacent vessels or cause dilation, congestion, or remodeling of nearby vessels, resulting in the Perilesional vascular sign on imaging (3). Nodule-in-nodule (or “island-like”) enhancement: this feature was less common; our index case exhibited classic “island-like” enhancement (**Figures 2A–D**), and similar findings have been sporadically reported (11, 15, 16, 18, 20, 21). Island-like enhancement is seen as scattered nodular enhancing areas within the solid portion of the lesion, resembling islands. This feature stems from intratumoral heterogeneity in GISTs—with variable cellular density, blood supply, and degrees of necrosis—where the “island” regions likely represent focal residual high-density active tumor tissue. To our knowledge, this sign has not been previously named, and we propose “island-like enhancement” as a novel, diagnostically valuable feature specific to pancreatic GIST.

The preoperative misdiagnosis rate of pEGISTs is extremely high, given that its imaging appearances overlap with various other pancreatic lesions. According to literature review, small solid pancreatic GIST (especially ≤5cm) should be distinguished from pNENs first, followed by SPNs in the differential diagnosis (56). pNENs are the second most common solid tumors after pancreatic ductal adenocarcinoma (PDAC) (57, 58) and often exhibit hypervascularity. Additionally, FDG-PET is valuable for evaluating metastatic spread and assessing the biological behavior of GISTs. Although ^68^Ga-DOTATATE PET is more specific for neuroendocrine tumors, it is occasionally utilized to differentiate GISTs from pNENs due to their overlapping hypervascular imaging characteristics (59, 60).Neither pNENs nor SPNs demonstrates the Perilesional vascular sign or island-like enhancement. Furthermore, SPNs typically occur in young women, have a well-defined capsule, exhibit progressive enhancement that does not exceed the degree of adjacent pancreatic parenchyma, and show no perilesional vascularity. For lesions >5 cm, the predominant presentation is cystic-solid masses with hyperenhancing solid components; differentiation from other mesenchymal tumors of the pancreas—especially hypervascular sarcomas such as leiomyosarcoma—is required. Although pEGISTs imaging characteristics (e.g., Perilesional vascular sign and island-like enhancement) may aid in distinguishing them from other pancreatic sarcomas, further study is needed to validate their diagnostic utility. It is important to emphasize that, regardless of a preoperative misdiagnosis, surgical resection remains the first-line treatment for pEGISTs and is unlikely to be delayed once imaging suggests a resectable hypervascular pancreatic mass. When pEGISTs exhibit multiloculated cystic-solid or predominantly cystic features, differentiation from MCNs and pancreatic pseudocysts becomes essential. Malignant-transformed MCNs often demonstrate elevated tumor markers (e.g., CA19-9), “eggshell” calcifications, mild enhancement of the solid components, and possible lymph node metastases; they do not exhibit perilesional vascularity. Pancreatic pseudocysts are rarely located within the pancreatic parenchyma per se and are typically associated with a history of pancreatitis. Consequently, a multimodality imaging approach is recommended for accurate preoperative diagnosis of pEGISTs, with careful consideration of the differential list.

From a pathological standpoint, CD117 and DOG1 are critical diagnostic markers for GISTs and EGISTs, with overwhelmingly high positivity rates. Recent studies indicate that combined testing for CD117 and DOG1 further enhances diagnostic sensitivity, especially in CD117-negative or atypical cases, where DOG1 serves as a valuable adjunct. It is crucial to note that pEGISTs characteristically have friable stroma; preoperative puncture biopsy, such as EUS-FNA or percutaneous core biopsy, may carry a risk of tumor seeding into the peritoneal cavity. Therefore, in cases with imaging findings highly suggestive of pEGISTs and definitive surgical intent, routine preoperative biopsy is not recommended.

Moreover, imaging plays an indispensable role in guiding treatment decisions. Contrast-enhanced CT can accurately assess tumor involvement of adjacent vascular structures and determine resectability, serving as a key component of preoperative risk stratification. MRI offers superior soft-tissue contrast and is advantageous in detecting hepatic metastases. PET-CT may be indicated to evaluate for distant metastatic disease and to inform postoperative adjuvant therapy and surveillance protocols.

In summary, pEGISTs are rare but clinically significant entity whose early identification depends on a comprehensive analysis of epidemiologic, radiologic, and pathologic features. Although its imaging characteristics lack absolute specificity, the presence of a well-circumscribed hypervascular pancreatic mass demonstrating marked heterogeneous enhancement, Marginal enhancement sign, Perilesional vascular sign, and/or island-like enhancement should prompt strong consideration of pEGISTs. Definitive diagnosis, however, ultimately relies on postoperative histopathology and immunohistochemistry. Future efforts should focus on larger, multicenter studies to refine the diagnostic pathway, develop standardized preoperative models for identification, and optimize management strategies to improve clinical outcomes and patient prognosis. Importantly, to minimize the risk of needle-track seeding and peritoneal dissemination, preoperative puncture biopsy is discouraged in cases where imaging suggests a resectable pEGISTs.

## Data Availability

The original contributions presented in the study are included in the article/**Supplementary Material**. Further inquiries can be directed to the corresponding authors.

## Supplementary material

Supplementary Figure 1 Contrast enhancement curve of the pancreatic lesion.
