# Supplementary figures and images for: Imaging features of pancreatic extragastrointestinal stromal tumors: a case report and literature review
Source: Front Oncol. 2025 Jul 29;15:1638850. doi: 10.3389/fonc.2025.1638850 (PMC12339331; doi:10.3389/fonc.2025.1638850)

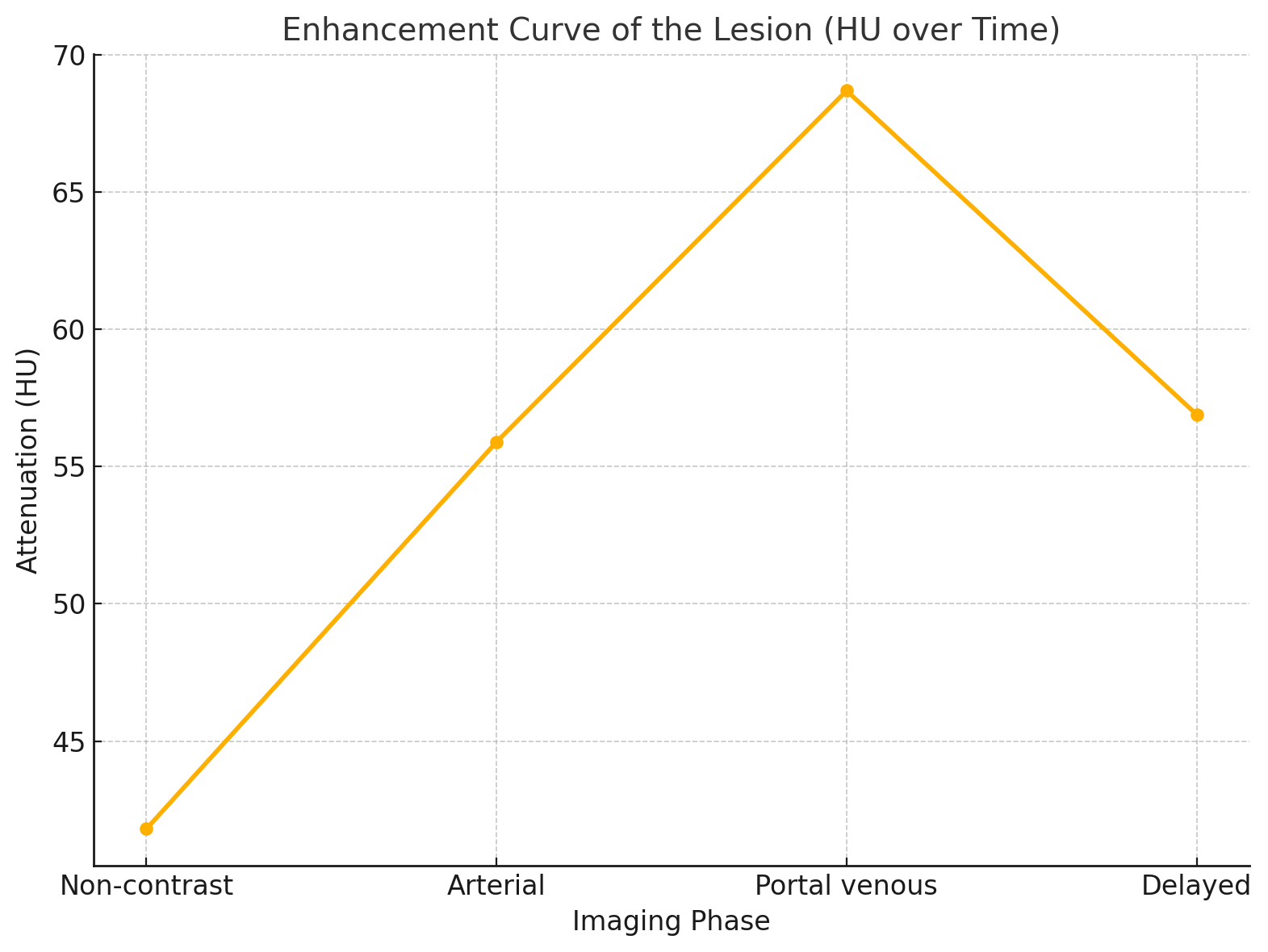

Supplement: Supplementary Figure 1 — Contrast enhancement curve of the pancreatic lesion. [file Image1.tif]
