# Supplementary material for: Imaging features of pancreatic extragastrointestinal stromal tumors: a case report and literature review
Source: Front Oncol. 2025 Jul 29;15:1638850. doi: 10.3389/fonc.2025.1638850 (PMC12339331; doi:10.3389/fonc.2025.1638850)
Supplement: Supplementary file 2 [file Table1.docx]

| **Supplementary Table 1**\| The clinical and imaging features of 50 patients were retrieved from the literature | | | | | | | | | | | | | | | | | | |
| --- | --- | --- | --- | --- | --- | --- | --- | --- | --- | --- | --- | --- | --- | --- | --- | --- | --- | --- |
| **Reference** | **Year** | **Age**  **(years)** | **Sex** | **Presentation** | **Tumor location** | **Tumor size(cm)** | **Component** | **Margin** | **Shape** | **Calcification** | **Necrosis** | **Enhancement types** | **Enhancement degree** | **Perilesional vascular sign** | **Island-like enhancement sign** | **Marginal enhancement sign** | **Imaging misdiagnosed lesions** | **NIH risk** |
| **Sharma et al.(7)** | **2024** | 62 | F | Abdominal pain | Body+Tail | 13.6 | cystic | well-defined | Oval | NA | YES | homogeneous | mild | YES | NO | YES | Pseudocyst | High |
| **Yin et al.(8)** | **2024** | 61 | M | Incidental finding | Head | 3.4 | Solid | Partially ill-defined | Round | NA | NO | heterogeneous | marked | YES | NO | YES | NA | NA |
| **Song et al.(9)** | **2024** | 51 | F | Incidental finding | Head | 5.7 | Solid | well-defined | Round | NO | YES | heterogeneous | marked | YES | NO | YES | NA | High |
| **Myralda et al.(10)** | **2023** | 46 | F | Abdominal mass | Body+Tail | 13.2 | Solid-cystic | well-defined | Round | NA | YES | heterogeneous | marked | YES | NO | YES | NA | High |
| **Beji et al.(11)** | 2022 | 53 | M | Abdominal pain | Body+Tail | 5.0 | Solid-cystic | Partially ill-defined | Round | NA | YES | heterogeneous | marked | YES | YES | NO | SPN | High |
| **Chen et al.(12)** | 2022 | 62 | M | Abdominal pain | Body+Tail | 22.0 | Solid-cystic | Partially ill-defined | Irregular | NO | YES | heterogeneous | mild | YES | NO | NO | MCN | High |
| **Zhao et al.(13)** | 2022 | 67 | F | Incidental finding | Head+Uncinate | 3.0 | Solid | well-defined | Oval | NO | NO | heterogeneous | marked | YES | YES | NO | pNENs | Low |
| Ene et al.(14) | 2021 | 53 | F | Malena+weight loss | Head | 8.0 | Solid | well-defined | Irregular | NO | YES | heterogeneous | mild | NO | NO | NO | NA | High |
| **Xie et al.(15)** | 2021 | 55 | M | Abdominal pain | Head | 3.4 | Solid | Partially ill-defined | Round | NO | YES | heterogeneous | marked | YES | YES | YES | NA | Low |
| **Xie et al.(15)** | 2021 | 61 | F | Incidental finding | Body+Tail | 18.9 | cystic -solid | well-defined | Irregular | NO | YES | heterogeneous | marked | YES | YES | YES | NA | High |
| **Yang et al.(16)** | 2019 | 54 | F | Abdominal pain | Head | 4.7 | Solid | Partially ill-defined | Round | NA | NO | heterogeneous | marked | YES | YES | NO | Malignant lesion | NA |
| **Duan et al.(17)** | 2019 | 57 | M | weakness | Head | 3.8 | Solid | well-defined | Round | NA | NO | heterogeneous | mild | YES | NO | NO | NA | Intermediate |
| **Tounsi et al.(18)** | 2018 | 49 | F | Abdominal pain | Head | 2.4 | Solid-cystic | well-defined | Oval | NA | YES | heterogeneous | mild | NO | YES | NO | SPN | NA |
| **Rasool et al. (19)** | 2018 | 60 | M | Abdominal pain+weight loss | Head | 10.0 | Solid-cystic | well-defined | Round | NA | YES | heterogeneous | marked | YES | NO | YES | NA | High |
| **Yol et al.(20)** | 2018 | 56 | M | Abdominal pain | Head | 10.5 | Solid-cystic | Partially ill-defined | Round | NA | YES | heterogeneous | marked | YES | YES | YES | NA | NA |
| **Yeo et al.(21)** | 2018 | 45 | F | Abdominal pain | Head+uncinate | 6.5 | Solid | well-defined | Oval | NO | NO | heterogeneous | marked | YES | YES | YES | pNENs | Low |
| **Xu et al.(22)** | 2018 | 42 | F | Abdominal pain | Head | 4.1 | Solid | well-defined | Oval | NO | NO | heterogeneous | marked | YES | NO | YES | SPN | Low |
| **Chi et al.(23)** | 2018 | 64 | M | Abdominal pain | Body+Tail | 28.0 | Solid-cystic | well-defined | Oval | NO | YES | heterogeneous | marked | YES | NO | NO | Malignant lesion | High |
| **Abderaheman et al.(24)** | 2017 | 53 | F | Abdominal pain | Head | 11.6 | Solid-cystic | Partially ill-defined | Irregular | YES | YES | heterogeneous | marked | NO | YES | NO | NA | High |
| **Kwon et al.(25)** | 2017 | 64 | F | Incidental finding | Head | 6.5 | Solid | well-defined | Round | NA | YES | homogeneous | marked | NO | NO | NO | pNENs | High |
| **Wu et al.(26)** | 2017 | 66 | M | Abdominal pain | Tail | 4.9 | Solid | Partially ill-defined | Oval | NO | YES | heterogeneous | mild | NO | NO | NO | Malignant lesion | Intermediate |
| **Liu et al.(27)** | 2016 | 56 | F | Incidental finding | Body | 5.7 | Solid-cystic | Partially ill-defined | Oval | NO | NO | heterogeneous | mild | YES | NO | YES | PDAC | NA |
| **Elgeidi et al.(28)** | 2016 | 30 | M | Abdominal pain+weight loss | Tail | 12.0 | cystic -solid | Partially ill-defined | Round | NA | YES | heterogeneous | marked | YES | NO | YES | NA | NA |
| **Xu et al.(29)** | 2016 | 61 | M | Abdominal pain | Body+Tail | 8.5 | Solid-cystic | well-defined | Oval | NO | YES | heterogeneous | mild | YES | NO | NO | Malignant lesion | High |
| **Aziret et al.(30)** | 2015 | 56 | M | Abdominal pain | Head | 4.0 | Solid | Partially ill-defined | Oval | NA | YES | heterogeneous | marked | YES | NO | YES | NA | High |
| **Xiao et al.(31)** | 2015 | 53 | M | Abdominal distension | Body+Tail | 12.7 | cystic -solid | Partially ill-defined | Oval | NO | YES | heterogeneous | mild | YES | NO | YES | MCN | High |
| **Lv et al.(32)** | 2015 | 54 | F | Incidental finding | Body | 4.6 | cystic -solid | Partially ill-defined | Irregular | NO | YES | heterogeneous | mild | YES | NO | NO | Malignant lesion | High |
| **Stanek et al.(33)** | 2015 | 55 | M | Incidental finding | Uncinate | 2.0 | Solid | well-defined | Irregular | NA | NO | heterogeneous | marked | YES | NO | NO | pNENs | High |
| **Tianet al.(34)** | 2014 | 61 | M | Incidental finding | Tail | 8.0 | Solid | well-defined | Oval | NA | NO | homogeneous | mild | NO | NO | NO | NA | Low |
| **Tianet al.(34)** | 2014 | 60 | M | Incidental finding | Head | 6.0 | Solid | Partially ill-defined | Round | NA | YES | heterogeneous | marked | YES | YES | YES | pNENs | High |
| **Akbulut et al.(35)** | 2014 | 61 | F | Weight loss | Head | 5.0 | Solid | Partially ill-defined | Round | NA | YES | heterogeneous | mild | NO | NO | NO | NA | Low |
| **Beltrame et al.(36)** | 2014 | 69 | F | Incidental finding | Uncinate | 2.4 | Solid | well-defined | Oval | NA | NO | heterogeneous | mild | YES | NO | YES | pNENs | High |
| **Ding et al.(37)** | 2014 | 61 | F | Chest distress | Tail | 12. 8 | cystic -solid | Partially ill-defined | Irregular | NO | YES | heterogeneous | mild | NO | NO | YES | Benign lesion | High |
| **Serin et al. (38)** | 2013 | 30 | M | Abdominal distension | Tail | 15.0 | cystic -solid | Partially ill-defined | Round | NA | YES | heterogeneous | marked | NO | NO | YES | Duplication cyst | High |
| **Soufi et al. (39)** | 2013 | 39 | M | Weight loss +abdominal pain+constipation | Head | 9.0 | Solid-cystic | Partially ill-defined | Round | NO | YES | heterogeneous | marked | YES | NO | NO | NA | Inermediate |
| **Wegge et al. (40)** | 2012 | 55 | M | Haematemesis+haematochezia | Head | 4.6 | cystic -solid | well-defined | Round | NA | YES | heterogeneous | mild | YES | NO | YES | NA | High |
| **Kim et al. (41)** | 2012 | 55 | M | Abdominal discomfort | Tail | 13.0 | Solid | well-defined | Irregular | NA | YES | heterogeneous | moderate | YES | NO | NO | SPN | Inermediate |
| **Cecka et al. (42)** | 2012 | 74 | F | Abdominal mass | Tail | 11.0 | cystic -solid | Partially ill-defined | Round | NA | YES | heterogeneous | marked | YES | NO | YES | NA | Intermediate |
| **Wang et al.(43)** | 2012 | 59 | M | Abdominal pain | Head | 5.5 | Solid-cystic | well-defined | Irregular | NA | YES | heterogeneous | marked | YES | NO | YES | NA | High |
| **Meng et al. (44)** | 2011 | 42 | M | Abdominal lump | Head | 10.0 | cystic -solid | well-defined | Round | NO | YES | heterogeneous | mild | NO | NO | YES | NA | Intermediate |
| **Wang et al. (45)** | 2011 | 55 | M | Abdominal distension | Body+Tail | 24.0 | cystic -solid | well-defined | Oval | NO | YES | heterogeneous | mild | YES | NO | NO | NA | High |
| **Zhu et al. (46)** | 2011 | 55 | M | Abdominal discomfort | Body+Tail | 30.0 | cystic -solid | Partially ill-defined | Round | NA | YES | heterogeneous | mild | YES | NO | NO | Pseudocyst | High |
| **Palhi et al. (47)** | **2010** | 42 | F | Weight loss+abdominal pain | Body+Tail | 35.0 | cystic -solid | Partially ill-defined | Irregular | NA | YES | heterogeneous | moderate | NO | YES | YES | NA | High |
| **Harindhanavudhi  et al .（48）** | **2009** | 63 | F | Fatigue+weakness+anemia | Body | 16.0 | cystic -solid | well-defined | Oval | NA | YES | heterogeneous | mild | NO | YES | YES | NA | High |
| **Trabelsi et al.(49)** | **2009** | 52 | F | Epigastric pain | Head | 10.5 | Solid | Partially ill-defined | Irregular | NA | YES | heterogeneous | marked | NO | NO | YES | NA | High |
| **Feng et al.(50)** | 2009 | 59 | M | Abdominal pain | Head | 12.0 | Solid-cystic | well-defined | Irregular | NO | YES | heterogeneous | marked | YES | YES | NO | Malignant lesion | High |
| **Yan et al. (51)** | **2008** | 47 | M | Nausea+vomiting | Uncinate | 2.4 | Solid | well-defined | Round | NA | NO | homogeneous | marked | YES | YES | NO | NA | NA |
| **Daumet al. (52)** | **2005** | 70 | F | Incidental finding | Head | 10.0 | cystic -solid | Partially ill-defined | Round | NO | YES | heterogeneous | marked | YES | NO | YES | NA | High |
| **Krska et al.(53)** | **2005** | 38 | F | Abdominal pain | Body | 17.0 | Solid | Partially ill-defined | Round | NA | YES | heterogeneous | mild | NO | NO | NO | NA | NA |
| **Yamaura et al.(54)** | **2004** | 54 | F | Incidental finding | Tail | 14.0 | Solid | Partially ill-defined | Irregular | NA | YES | heterogeneous | marked | YES | NO | NO | NA | Low |
| Solid-cystic:Predominantly solid with cystic component  cystic -solid:Predominantly cystic with peripheral solid component  pNENs:Neuroendocrine tumors  SPN:Solid pseudopapillary neoplasm  MCN:Mucinous cystic neoplasms  PDAC:Pancreatic ductal adenocarcinoma  NIH：National institutes of health  NA：Unable to assess | | | | | | | | | | | | | | | | | | |
